# Understanding and retention of the informed consent process among parents in rural northern Ghana

**DOI:** 10.1186/1472-6939-9-12

**Published:** 2008-06-19

**Authors:** Abraham R Oduro, Raymond A Aborigo, Dickson Amugsi, Francis Anto, Thomas Anyorigiya, Frank Atuguba, Abraham Hodgson, Kwadwo A Koram

**Affiliations:** 1 Navrongo health research centre, Ghana health service, Box 114, Navrongo, Ghana; 2 Noguchi memorial institute for medical research, University of Ghana, Box LG 581, Accra, Ghana

## Abstract

**Background:**

The individual informed consent model remains critical to the ethical conduct and regulation of research involving human beings. Parental informed consent process in a rural setting of northern Ghana was studied to describe comprehension and retention among parents as part of the evaluation of the existing informed consent process.

**Methods:**

The study involved 270 female parents who gave consent for their children to participate in a prospective cohort study that evaluated immune correlates of protection against childhood malaria in northern Ghana. A semi-structured interview with questions based on the informed consent themes was administered. Parents were interviewed on their comprehension and retention of the process and also on ways to improve upon the existing process.

**Results:**

The average parental age was 33.3 years (range 18–62), married women constituted a majority (91.9%), Christians (71.9%), farmers (62.2%) and those with no formal education (53.7%). Only 3% had ever taken part in a research and 54% had at least one relation ever participate in a research. About 90% of parents knew their children were involved in a research study that was not related to medical care, and 66% said the study procedures were thoroughly explained to them. Approximately, 70% recalled the study involved direct benefits compared with 20% for direct risks. The majority (95%) understood study participation was completely voluntary but only 21% recalled they could withdraw from the study without giving reasons. Younger parents had more consistent comprehension than older ones. Maternal reasons for allowing their children to take part in the research were free medical care (36.5%), better medical care (18.8%), general benefits (29.4%), contribution to research in the area (8.8%) and benefit to the community (1.8%). Parental suggestions for improving the consent process included devoting more time for explanations (46.9%), use of the local languages (15.9%) and obtaining consent at home (10.3%).

**Conclusion:**

Significant but varied comprehension of the informed consent process exists among parents who participate in research activities in northern Ghana and it appears the existing practices are fairly effective in informing research participants in the study area.

## Background

Informed consent as a process, enables persons to voluntarily decide whether or not to participate in a research study or procedure. The process ensures respect for individual autonomy to take part in a research activity. It is more than merely getting a potential participant to sign or thumbprint a written document to indicate the legal basis for consent and for future reference [1-3]. The signing of a consent document begins a process of deliberations between the research team and participants, which enables them to decide whether to continue in the research study or not. The process is expected to be updated if any new information emerges and to ensure that participants have the opportunity to ask questions and raise concerns before, during, and even after the study. The process is therefore continuous and interactive rather than a one-time information session [1,3].

The challenge, however, remains as to whether many participants do comprehend the basic information provided regarding the research they are asked to take part in. Evidence suggests that many participants have varied appreciation of the study in which they are enrolled or know their rights as participants [4-6]. Indeed, to some, any research in which the economically disadvantaged dominate is in reality ethically problematic as they are vulnerable to exploitation and impaired decision-making [7]. However, the objective to generate generalizable results and to fairly distribute risks and benefits of research, oblige researchers not to bar underprivileged persons as participants without cause. Thus, while it is desirable to have a recognized standard of informed consent, the debate is whether research ethics should not be adapted to suit the culture and other socio-economic distinctiveness of the study population [1,3].

To adequately address some of these challenges, the process must provide effective communication, sufficient information and a high level of comprehension [6,7]. Other considerations should be based primarily on the manner in which the details of the consent process are presented [7]. The use of visual aids for instance, can improve the participants' ability to remember facts much better than verbal presentations. Furthermore, designing and administering the informed consent document in a manner that pay special attention to the vulnerable and those with special needs is essential [7-13]. In addition, other inter-related issues including conceptual and linguistic barriers to effective communication and other socio-economic factors must be taken into cognisance in the process [12,13]. The language, both verbally and written, and the presentation of the various themes must be understandable to an average participant [8-12]. As a process, the consent document must also be revised when deficiencies are noted or new information becomes available during the research. This may include reminders to aid in deciding whether to continue participation as the idea of making an informed decision may often be compromised initially when participants may not have immediate alternatives to the benefits to be derived from the research [6,10-12]. Though participants may frequently not understand information disclosed to them in the consent process, no standard exists as to how this can be improved markedly. Moreover, efforts at improving understanding through the use of other medium of communication and enhanced consent forms have had varied successes. Having a study team member or a neutral educator spend more time talking one-on-one to participants appears to be one of the most effective ways of improving the process [10,13,14].

As a rule in the Navrongo Health Research Centre, all research studies require approval of the consent document by the ethics committee and witnessed informed consent from potential participants before enrolment. To date, however, there has not been any systematic follow up to evaluate the process and determine the level of comprehension despite several interventions taking place in this setting [15-17]. The study was therefore designed to determine parental comprehension and retention of the consent process as part of evaluation of the existing practices.

## Methods

### Study setting

The study was conducted in the Kassena-Nankana District (KND) of Ghana. This rural area exemplifies many of the socio-economic challenges of tropical Africa. The district has a population of about 150,000 people and the geography reflects that of the Guinea savannah. Two ethno-linguistically distinct groups, the Kassenas and the Nankanis, populate the district. They have similar socio-cultural institutions such as the extended family system and patrilineal inheritance. Traditions of marriage, kinship and family building underscore the security and economic values of the people. The economy of the area is dominated by subsistence agriculture. Literacy rate is low, particularly among women and the people are mostly Christians. Health-seeking behaviour is sometimes governed by tradition rather than modern health care [18-20]. The Navrongo Health Research Centre is an institution of the Ghana Health Service, which was established in the late eighties to conduct research and to test primary health care strategies to inform policy. It conducts broad-based research activities involving the biomedical, social sciences, as well as other research on critical national health issues. Since its inception, there has been several intervention trials conducted in the prevailing context of high morbidity, poverty and adversity, thus making research ethics issues a great challenge in the setting [15-17].

### The cohort study design

From June 2005 to June 2006, a total of 330 healthy children were enrolled and followed up in a malaria cohort study that evaluated the immune correlates of protection against childhood malaria. Participants (parents of children aged 1 – 5) were selected randomly from the Navrongo Demographic Surveillance System (NDSS) and approached for enrolment. At the beginning of the study and for every two months during the subsequent twelve months, the participants were contacted by the study team to interview the parents and conduct study related procedures. These involved physical examination by study clinicians and finger prick blood specimen for the detection of malaria parasites and estimation of haemoglobin concentration. In addition, about 0.5–1.0 millilitre of whole blood was taken from the finger every two months for the analysis of antibodies against malaria. Among the benefits for participating in the study were frequent examination for illnesses and free treatment of febrile illness during the study. In addition, the study fieldworkers, living in the communities of the participants, facilitated contact with the study team and assisted with transportation to health facilities where necessary. Adding to the direct benefits, participants were told that the children were contributing to the ultimate aim of finding a vaccine for malaria. On the part of risks, the frequent examinations posed inconvenience to the participants and their families. The children could also experience pain whenever blood was taken through pricking their fingers. There was also the remote possibility of the site of the finger pricks becoming infected and the possibility of excessive bleeding from these sites. At the end of the cohort study, the parents were interviewed to determine their understanding and retention of the study informed consent process.

### The cohort study consent process

The consent document was written in English, translated into the two major local languages (Kasem and Nankani) and back translated into English. The local ethics committee approved all translations and back translations. The consent document was structured as follows; a title and sections comprising introduction to the study, study procedures, inclusion and exclusion criteria, risks and benefits of the study, confidentiality, right to withdraw, compensation, persons to contact for questions and further clarification and signature. Parents had verbal disclosure of information to them from trained field workers who had high school grade certificates and who understood the local languages. The field workers were trained and made to pilot the consent document before administering it at enrolment. On the average, about thirty minutes was taken to explain the study to each mother.

### The consent study design

All mothers who consented in the cohort study were eligible for the consent study and were contacted. All the parents who were available and consented to take part in the informed consent study were interviewed. This study used semi-structured questionnaire with questions based on the key themes of the parental informed consent form used during the cohort study. In addition, explanation or comments that parents had for the interviewers concerning the parental consent processes and procedures were captured. After the necessary ethical approvals, the field staff were trained on the objectives of the ethics study and the study instruments, and were made to pilot the study instruments to correct all inconsistencies. This was to ensure that our understanding of the questions was the same as the interviewers' and the respondents' and also to make sure that translating and asking the questions in the local dialects and recording of the responses from the interviewees were comparable. The parents were then informed about the study and appointments made for the interviews at their convenience. The questionnaire was administered to the parents at their homes. The questionnaire was designed for a major question to be asked on each theme to determine whether information was given to the mother on that theme during the cohort study. Sub-questions were then asked to ascertain the parent's appropriate recollection of the information that was disclosed. For instance, participants were asked if they knew the previous study involved risks and if yes, they were asked to mention some of the risks. Data collection took place within a ten-day period to ensure there was no diffusion of responses from the already interviewed respondents to those yet to be interviewed. Every parent was interviewed once irrespective of the number of children she had on the cohort study.

### Data analysis

After completion of data collection, the data were reviewed for inconsistencies and where necessary, corrections were made after contacting the respondents before data entry and analysis were done. For the open-ended questions, two research assistants reviewed all the responses, coded and entered them into a database. Where there were disagreements between them, a third opinion was sought and a consensus arrived at. Data were double entered into Epi info (version 2003) database and analysis done using Stata (version 7). Findings are presented as tables, graphs and texts. The statistical tests planned were student's t tests for all continuous variables and Chi-square tests for categorical variables. Point estimates are computed as means, proportions or percentages for all the background characteristics, informed consent themes and other variables measured in the study. Interval estimates are in 95% confidence intervals and ranges. All statistical tests were two sided and an alpha level ≤ 0.05 was considered significant.

### Ethical issues

The main justification for the study was to generate information that will help improve upon the way informed consent is obtained from potential research participants. The study did not present any direct benefit or risk to the participants. However, we obtained informed consent from all the participants and ethical approval from the Navrongo Health Research Centre Institutional Review Board.

## Results

### Baseline characteristics

In all, 300 parents whose children participated in the cohort study were contacted. Of these, 90% (270/300) granted consent and were interviewed. Of the remaining 30; nineteen (19) had travelled outside the study area during the interview period and eleven (11) refused to participate. Participants were all females; about 96% (259/270) were biological parents with three as the median number of children alive. Approximately, 15% (95% CI 10.7, 19.6) of the respondents were young parents (<25 years) and 26.7% (95% CI 21.4, 32.4) were over forty years. The average age was 33.3 years (Range 18–62). They were mostly Christians; 72% (194/270), married couples; 92% (248/270) and farmers; 62% (168/270). About 53% (95% CI 46.8, 59.0) had no formal education and 42.6% (95% CI 36.7, 48.7) had only basic level (9 years) education (details in Table 1). Majority, 94.4% (255/270) were from the two major ethnic groups: [Kassenas, 58.9% (95% CI 52.7, 64.8) and Nankanis 38.9% (95% CI 33.0, 45.0].

**Table 1:** Baseline socio-economic characteristics of study participants

**Characteristics**	**Categories**	**% (n)**	**[95%CI]**
Age groups (years)	< 25	14.8 (40)	[10.7, 19.6]
	25–35	48.5(131)	[42.4, 54.6]
	> 35	36.7(99)	[30.9, 42.7]
Formal education (years)	None	53.7 (145)	[47.5, 59.8]
	1–9	42.6 (115)	[36.6, 48.7]
	> 9	3.7 (10)	[1.7, 6.7]
Primary occupation	Farming	62.2 (168)	[56.1, 68.0]
	Trading	34.1 (92)	[28.4, 40.1]
	Others	3.7 (10)	[1.7, 6.7]
Marital status	Married	91.9 (248)	[87.9, 94.8]
	Single	2.6 (7)	[1.0, 5.3]
	Others	5.6 (15)	[3.1, 9.0]
Religion	Christianity	71.9 (194)	[66.0, 77.1]
	Islam	0.7 (2)	[0.1, 2.1]
	African traditional	27.4 (74)	[22.1, 33.1]

The table shows the various categories of baseline socioecominc characteristics of the study participants.

### Correlation of characteristics

Baseline features categorized into respondents' age groups (<25 or ≥ 25 years), educational level (some or never) and type of occupation (farming or others) were correlated with selected themes (Table 2). In all, younger parents had more consistent understanding of the consent process than the older ones. For instance, parents under 25 years were twice more likely to remember study risks than those over 25 years (35.0% vs. 17.8%; p-value = 0.01). Similar findings were found for age with other themes like study procedures (80.0% vs. 63.9%) and benefits (85.0% vs. 66.1%). However, correlating parental education and occupation to the same themes did not show any consistent significant differences (Table 2).

**Table 2:** Relation between parental characteristics and understanding

Characteristics	**Age group (ys), %**	**Formal Education, %**	**Occupation, %**
	< 25 (40) vs. >24 (230)	Some (125) vs. Never (145)	Farming (168) vs. Others (102)
**Study Procedure**	80.0 vs.63.9	70.4 vs. 62.8	63.7 vs. 70.5
Diff. (95% CI) *p-value*	16.1 (2.2, 30.0) p = 0.04*	7.6 (-3.6, 18.5) *p = 0.18*	6.8 (-18.2, 4.7) *p = 0.25*
**Selection criteria**	70.0 vs. 62.2	65.6 vs. 61.4	67.9 vs. 55.9
Diff. (95% CI) *p-value*	7.8 (-7.7, 23.3) *p = 0.34*	4.2 (-7.2, 15.7) *p = 0.47*	12.0 (0.1, 23.9) p = 0.0*
**Study benefits**	85.0 vs.66.1	72.4 vs. 64.1	66.7 vs. 72.5
Diff. (95% CI) *p-value*	18.9 (6.2, 31.5) p = 0.01*	8.3 (-2.7, 19.4) *p = 0.14*	5.8 (-17.0, 5.4) *p = 0.318*
**Study risk**	35.0 vs.17.8	21.6 vs. 19.3	15.5 vs. 28.4
Diff. (95% CI) *p-value*	17.2 (1.6, 32.8) p = 0.01*	2.3 (-7.3, 12.0) *p = 0.63*	12.9 (-23.2, -2.6) p = 0.0*

*Statistically significant findings

A table showing association between categories of parental characteristics and the percentage of them who had comprehension of selected informed consent themes.

#### Informed consent themes

##### Introduction

**Figure 1:**
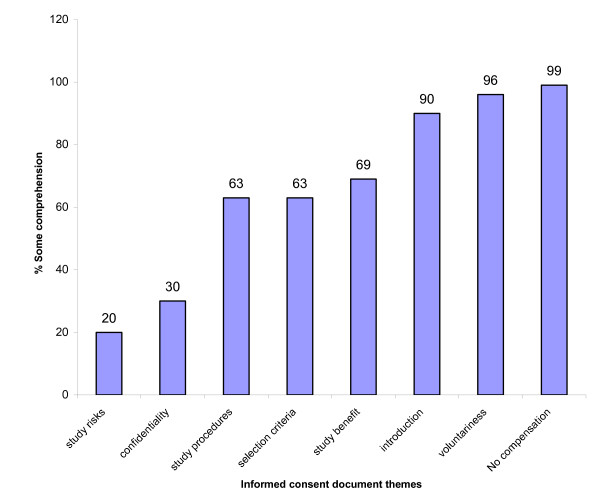
**Bar chart of parental comprehension of research informed consent**. The Bar chart shows the percentage of parents who gave responses to indicate they understood information given under a particular informed consent theme.

Over 90% (264/270) of the parents interviewed knew that their children were participating in a research study of the Navrongo Health Research Centre and not for direct medical care. However, only 23% (95% CI 18.0, 28.4) said the study involved other collaborators. About 90% (245/270) said the disease the research was associated with was malaria (Figure 1). Most parents 63.3% (171/270), knew the children were enrolled using a selection criteria and 70.8% (95% CI 63.3, 77.5) of them said they were told the reasons why some children were not to be enrolled. When asked to mention reasons why some children were not enrolled, 72.7% (95% CI 63.8, 80.40) of 121 parents who said they were told the reasons why some children were not to be enrolled, could mention at least one of such reasons correctly. Again, 70.7% (191/270) remembered they were told the total number of children to be enrolled but only 20.4% (95% CI 15.7, 25.7) knew correctly that the study involved children less than five years of age.

##### Study procedures

Approximately two-thirds of parents 66% (179/270), admitted the study procedures were explained to them during the consenting process and 93.8% (168/179) could mention at least one procedure correctly. When asked specifically about the duration of the study and frequency of contacts, 79.3% (214/270) and 78.1% (211/270) respectively, answered correctly. Only about 40% (120/270) did remember the amount of time they were to spend during the study. Again, 92% (249/270) remembered that blood specimen would be taken from the upper arms but only 55% (149/270) knew one correct reason for taking the blood specimen.

##### Risks and benefits

The study documented significant differences between the proportion of respondents who recalled that they were told the study involved direct benefits 69% compared with direct risks 20.4% (48.6% 95% CI 41.2, 55.9; *p-value < 0.001*) Figure 1. About 95% (176/186) who recalled being told about direct benefits could mention at least one direct benefit to the study child correctly. Of the 55 participants who recalled being told about study risks, 87% (48/55) could mention at least one risk correctly, 45.5% (25/55) recalled the steps that were to be taken to minimize such risks and 24% (6/25) could mention at least one of such steps correctly. Again, a significant difference was documented between those who recalled receiving information about both benefits and risks and those who did not; 25.9% versus 15.2% (10.7% 95% CI 03.9, 17.5 *p-value < 0.002*). Also, 53.7% (145/270) recalled being given information on only benefits compared to 5.2% (14/270) on only risks. All except one respondent (269/270) remembered that participants would not be paid for study participation.

##### Withdrawal

Approximately, 96% (259/270) admitted being told that the study participation was completely voluntary and about 62% (169/270) believed that nothing would have happened to them or their children if they had refused to let their children participate in the study. However, only 21% (57/270) remembered being told that they could withdraw their consent to allow their children to participate in the study at anytime without giving reasons. Of this, 62.2% (36/57) said they believed what the investigators told them. Only 36.7% (99/270) recalled that the children could be withdrawn by the investigators if it was found necessary to do so.

##### Confidentiality

About 30% (82/270) remembered that they were told the information collected in the study will be used only for the purpose for which consent was obtained and only 7.8% (21/270) remembered that the specimen collected will not be used for any other study without appropriate permission. Approximately, 37% (100/270) recalled being told that the participants' data will be safely kept in confidence by the investigators.

##### Research participation

Among the respondents, about 3% (95% CI 1.0, 5.3) had ever participated in a research study conducted by the research Centre, while about 53.7% (95% CI 47.6, 59.8) had had at least one child or relation ever participate in a research study conducted by the research Centre. About 16% (95%CI 12.1, 21.3) of the respondents had a relation working with the research Centre. Among some of the reasons given by 170 respondents who gave reasons for allowing their children to participate in the research work were; free medical treatment 36.5% (95% CI 29.2,44.2), general benefits to the participant 29.4% (95% CI 22.6, 36.9), better medical care 18.8% (95% CI 13.2, 25.5), contribution to the research centre's work 8.8% (95% CI 5.0,14.1), benefit of the study to the local community 1.8% (95% CI 0.3, 5.1) and other benefits 4.7% (95% CI 2.0, 9.1). When participants were asked to express their views on how the informed consent process could be improved, 53.7% (145/270) made suggestions like devoting more time for explanations (46.9%), use of the local languages (15.9%) and obtaining consent at home (10.3%). Other details in Table 3.

**Table 3:** Participants suggestions for improving the informed consent process.

**Parental suggestions (n = 145)**	**% (n)**	**[95%CI])**
1. Devote more time to explain the study	46.9 (68)	[38.5, 55.3]
2. Explain the consent in local languages	15.9 (23)	[10.3, 22.8]
3. Explain the study in patient homes	10.3 (15)	[5.9, 16.4]
4. Investigators should decide the best way	7.6 (11)	[3.8, 13.1]
5. The need to reconsent after some time	6.2 (9)	[2.8, 11.4]
6. Other suggestions	13.1 (19)	[8.0, 19.7]

A table of suggestions and ways made by the study participants to the investigators to help improve the informed consent process in the study area.

## Discussion

Any conduct of human research that promotes a climate consistent with high ethical standards should reflect the informed consent process since that is where the basis of the research, procedures involved, inherent benefits, procedural risks, and for patients; the therapeutic alternatives are disclosed [1,7,22,23]. To date, however, major challenges confront several aspects of the process despite several guidelines that highlight the importance of obtaining consent and practical steps to guide the process. The difficulty often hangs on how to guarantee the sanctity of the process and to ensure that potential participants truly understand the research they are invited to participate in. Indeed, the available ethical guidelines only provide a framework for research practice rather than providing comprehensive regulations within which researchers should act or be regulated and as such does not necessarily guarantee good scientific and professional decisions [1-3]. The need for self-evaluation of consent procedures before, during and after every research activity in addition to those provided by the ethics committees, in order to address any emerging challenges cannot be over emphasized [3-5].

This study evaluated comprehension and retention of study information in a cohort of parents who had enrolled their children in a malaria study in northern Ghana. A significant number of research studies that present ethical challenges, including informed consent, have been conducted in this area. Given that common ethical situations remain unfamiliar to many of the potential participants in the study setting, appreciation of those situations is a necessary first step in responding well to them [20,21]. Understanding as a concept model is very difficult to conceptualise let alone measure even among literate populations [12,22-24][33]. The assumption made in this study is that knowledge, in its simplest form, is some awareness of information administered and that this knowledge correlates with understanding. One way or the other, the decision whether to participate in a research study or not cannot be seen to be 'correct or otherwise'.

The results of the study showed significant but varied knowledge in the informed consent process in relation to the various consent themes used to evaluate the participants. Most of the respondents had a good grasp of the introductory aspect of the consent document but there were mixed outcomes with respect to different ethical concepts. Majority of the parents interviewed knew that their children were participating in a research on malaria that was not directly related to medical care and that not all children were to be enrolled due to a predetermined selection criteria. This is a very significant finding, not only because an unexpected number of parents could retain the information after a year but also because it is at variance with results of other reported studies which suggests that many parents have poor understanding of study information particularly among those who are poor and have lower education [4,10-12]. A possible explanation for the findings, however, could be due to the fact that the study area has been under intensive research for many years with several major interventions having been conducted [15-17]. Thus, the inhabitants could have over the years gotten use to the process of informed consent and the activities of the research Centre in contrast to other settings where this kind of situation has not existed. There may therefore be the need for similar evaluations to be undertaken in comparable settings where research activities have been very prevalent to compare with this finding.

Also, a careful assessment of some of the misgivings about the informed consent process and its understanding especially in less endowed areas, reveals that most of the concerns are easily resolved by improving the existing process and the manner in which the facts are presented to participants, because the ability to recall facts is based on these factors. In recent times, even in resource poor settings, informed consent documents are designed and administered in a manner that pay special attention to the vulnerable; all consent documents including the translations are approved by the host institution's ethics committee and those who administer the documents are often persons who are research team members and understand the local language, customs and norms of the potential participants. They usually spend time talking to potential study participants to ensure adequate understanding before enrolment.

While it may be true that in the past, understanding of informed consent in less endowed countries was low, these countries are never static and homogenous in nature. Moreover, progress in research ethics has improved tremendously in recent years due to increased awareness and capacity building activities. Indeed, improvement in Good Clinical Practices (GCP) and ethics training in many low-income countries are contributing to understanding of research procedures in many areas. Agencies like the National Institutes of Health (NIH), the African Malaria Network Trust (AMANET) and the World Health Organization (WHO) have been conducting capacity building workshops in research ethics for review committees and investigators and this is helping to solve some of the existing ethical issues in many of these resource poor settings [25,26].

One area in which the study recorded low comprehension among respondents was related to ethical procedures and terms whose meaning have always been difficult to communicate. For instance, less than a third of respondents understood that the information collected in the study would be kept in confidence. Again, fewer than expected number of respondents in the study knew the reasons for which their biological specimen was taken. These findings, though at odds with other findings, reflect the difficulties in translating medical terminologies into lay and local languages, and in some cases dialects [23]. Sometimes, informed consent can be intricate to administer in these settings, as translation of concepts from English into the local languages remain a major problem. In many parts of the world, official languages are those of the former colonial powers and though many people speak their native languages, only a few can read and write these languages. How does an illiterate understand an ethical concept he or she cannot locally communicate using it, especially concepts that he or she is not familiar with or has not heard before? One is able to adequately comprehend a term or a word if one can consciously replicate the information conveyed by it in his or her local language.

The study further showed the existence of significant differences between the proportion of respondents that recalled study benefits compared with risks (69% versus 24.4%). This finding may be interpreted in two ways; either research participation in this setting is influenced by trust and beneficial outcomes or the mothers recalled more of the benefits than risks due to the fact that the risks involved in the cohort study were minimal; blood draws and questions every other month compared with free diagnosis and treatment of acute illnesses for one year. Whichever way this issue is viewed, the question that arises is whether it matters to participate in a research for the anticipated benefits given the fact that the participants did understand the study and with their prevailing health needs they decided to participate. Previous studies in this setting have revealed that trust, social norms and others rather than information disclosed through the consent process may be what influence participation in research [20,21]. This is because in many rural communities, the implicit trust engendered in the long term relationship between the research team and the community may be more important than signing an informed consent document. The challenge then will be how to balance the requirements of the international ethics research community and the local norms and expectations.

Furthermore, younger parents had considerable understanding of the consent process than the older ones. Younger parents were more likely to remember study risks than their older counterparts probably due to the fact that the younger parents were better educated or that both the old and young adults equally understood the information initially but retention among the older adults' declined faster than the young adults. Again, the question as to how much information is required for it to be considered 'informed' is yet to be fully addressed. Should the amount and level of information given be dictated by the participants' socio-economic level, the type of research or by the researcher? While some participants would not want to be burdened with all the details of the research study, others sometimes require an in depth understanding before they can make a decision. The amount of information available to a participant at a particular time can influence the decision to participate in research. For those already in a study, such information should be capable of making the participant rescind his or her earlier decision and withdraw. Clearly, however, this may be impossible in many areas unless there were periodic reminders to enable those who want to rescind their decisions do so without fear or favour.

Other relevant findings from the study include suggestions made by the respondents on how to improve the informed consent process. Crucial among them was the need for individuals administering the consent document to spend more time in doing so, which may be extremely difficult in studies with large sample sizes. Others were that consent should be sought at the potential participant's home and should be conducted in the local language. These are all accepted factors known to enhance the process. What was striking, however, was the idea that the investigators should decide the best way, engendering a possible reflection of the trust discussed earlier.

## Study limitations

Among the limitations of this study are the difficulties in conceptualising understanding and its measurements especially as it relates to ethical concepts. Another potential limitation may be the difficulty in a parent's recall of study information disclosed to her over a year ago. Also, the ability to retain the information might not necessarily imply that full understanding was indeed achieved as measured in the study. Being a study conducted among women with young children, the findings may not be generalizable to the whole community.

## Conclusion

The study showed that significant but varied comprehension of information disclosed during the informed consent process exists among parents who participate in research conducted by the Navrongo Health Research Centre. Thoughthe existing practices appear to be fairly effective in informing research participants in the study area, there is the need for further studies and similar evaluations in related settings to compare with this study and to clarify some of the findings. There is also the need to continue to evaluate the existing practices to improve upon the standards achieved so far.

## Competing interests

The corresponding author has full access to all the data in the study and had final responsibility for the decision to submit for publication in this journal. All authors declare that they have no conflict of interest whether financial or non-financial.

## Authors' contributions

ARO, RAA, DA, FA, FA, TA. Conception, design, data acquisition and interpretation. Also they contributed to manuscript drafting and revision for the intellectual content. AH, KAK. Design, data interpretation, and manuscript preparation and revision for the intellectual content.

## Pre-publication history

The pre-publication history for this paper can be accessed here:


